# Vitamin D and Clinical Cancer Outcomes: A Review of Meta‐Analyses

**DOI:** 10.1002/jbm4.10420

**Published:** 2020-11-04

**Authors:** John D Sluyter, JoAnn E Manson, Robert Scragg

**Affiliations:** ^1^ School of Population Health, University of Auckland Auckland New Zealand; ^2^ Department of Medicine Brigham and Women's Hospital, and Harvard Medical School Boston MA USA; ^3^ Department of Epidemiology Harvard T.H. Chan School of Public Health Boston MA USA

**Keywords:** CANCER INCIDENCE, CANCER MORTALITY, CIRCULATING 25‐HYDROXYVITAMIN D, META‐ANALYSIS, RANDOMIZED CONTROLLED TRIAL, VITAMIN D

## Abstract

The relationship between vitamin D status or supplementation and cancer outcomes has been examined in several meta‐analyses. To address remaining knowledge gaps, we conducted a systematic overview and critical appraisal of pertinent meta‐analyses. For meta‐analyses of trials, we assessed their quality using AMSTAR‐2 (A Measurement Tool to Assess Systematic Reviews), strength of associations using umbrella review methodology and credibility of evidence using GRADE (Grading of Recommendations, Assessment, Development, and Evaluation) criteria. Meta‐analyses of observational studies reported inverse associations of 25OHD with risk of cancer incidence and cancer mortality and, particularly for colorectal cancer, fulfilled some of Bradford‐Hill's causation criteria. In meta‐analyses of trials, vitamin D supplementation did not affect cancer incidence. However, we found credible evidence that vitamin D supplementation reduced total cancer mortality risk, with five out of six meta‐analyses reporting a relative risk (RR) reduction of up to 16%: RR, 0.84 (95% CI, 0.74–0.95). The strength of the association, however, was classified as weak. This was true among meta‐analyses of high, moderate, and lower quality (AMSTAR‐2–rated). Trials did not include large numbers of vitamin D‐deficient participants; many tested relatively low doses and lacked sufficiently powered data on site‐specific cancers. In conclusion, meta‐analyses show that, although observational evidence indicates that low vitamin D status is associated with a higher risk of cancer outcomes, randomized trials show that vitamin D supplementation reduces total cancer mortality, but not cancer incidence. However, trials with larger proportions of vitamin D‐insufficient participants and longer durations of follow‐up, plus adequately powered data on site‐specific common cancers, would provide further insight into the evidence base. © 2020 The Authors. *JBMR Plus* published by Wiley Periodicals LLC on behalf of American Society for Bone and Mineral Research.

## Introduction

Ecological studies of cancer incidence and mortality have shown that sun exposure, especially solar UV B (and hence vitamin D production), is associated with reduced risk of many cancer types.^(^ ^1^, ^2^, ^3^ ^)^ This supports the hypothesis that vitamin D may have a beneficial impact on cancer outcomes, which is further strengthened by the identification of biological mechanisms that may explain these associations^(^ ^4^ ^)^ and prospective observational studies, which show that dietary intake of vitamin D (including vitamin D supplementation) is associated with a reduced risk of cancers, although confounding is important to consider.^(^ ^5^, ^6^, ^7^, ^8^ ^)^

Meta‐analyses provide accurate, succinct, credible, and comprehensive summaries of the best available evidence, and act as a key tool for health care professionals to achieve evidence‐based decisions.^(^ ^9^ ^)^ Several meta‐analyses have synthesized data on the association of vitamin D status with cancer outcomes from observational studies^(^ ^5^, ^6^, ^7^, ^10^, ^11^, ^12^, ^13^, ^14^ ^)^ and the effect of vitamin D supplementation on cancer outcomes in randomized clinical trials.^(^ ^15^, ^16^, ^17^, ^18^, ^19^, ^20^, ^21^, ^22^ ^)^ However, the evidence base is inconsistent and fragmented into various meta‐analyses that combine different study populations and outcomes, making assessment of the evidence using a similar methodological framework difficult. Further, as the methodologic quality of vitamin D–cancer meta‐analyses may vary, uncritically accepting their results carries risk.^(^ ^9^ ^)^

Thus, we decided to conduct a systematic overview and critical appraisal of pertinent meta‐analyses to better characterize the evidence on vitamin D status or supplementation in relation to cancer outcomes. In our critical appraisal, particular attention was given to intervention studies as this study design, as the gold standard for effectiveness research, provides the highest relevance for evidence‐based decision‐making. For this, we systematically assessed the quality of meta‐analyses, plus strength and credibility of the evidence from these studies across multiple cancer outcomes, and discussed differences between meta‐analyses. Finally, we discussed limitations of the evidence presented and provided some future research directions.

## Materials and Methods

We searched Medline and PubMed for articles published up until 12 May 2020, using the following search terms: vitamin D, cancer, and meta‐analysis. No language restrictions were applied. This was supplemented by a manual search of reference lists from identified articles.

The quality of meta‐analyses was assessed using AMSTAR‐2 (A Measurement Tool to Assess Systematic Reviews), a 16‐point assessment tool of the methodological quality of systematic reviews.^(^ ^9^ ^)^ Of the 16 domains, items 2, 4, 7, 9, 11, 13, and 15 are considered “critical” domains (can critically affect the validity of a review and its conclusion).^(^ ^9^ ^)^ Based on weaknesses in the critical and noncritical domains, the overall confidence in the results of the meta‐analysis was classified as “High,” “Moderate,” “Low,” or “Critically low.”^(^ ^9^ ^)^ AMSTAR‐2 has good interrater agreement, test–retest reliability, and content validity.^(^ ^9^ ^)^

The strength of associations was evaluated based on umbrella review criteria.^(^ ^23^ ^)^ For this, small‐study effects were evaluated using Egger's test,^(24^ ^)^ and the excess significance test was applied (excess significance was claimed at *p* ≤ 0.10).^(^ ^25^, ^26^ ^)^ Based on these criteria findings, we classified the strength of the association (effect) as “Convincing,” “Highly suggestive,” “Suggestive,” or “Weak” (Appendix Table AA1).^(^ ^23^ ^)^ Associations were considered nonsignificant if the *P* value was >0.05. To provide a practical metric of the efficacy of vitamin D supplementation, the number needed to treat (NNT) was calculated.^(^ ^27^ ^)^

**Table A1:** Umbrella Review Assessment Grades

Strength of association	Criteria
Convincing (class I)	>1000 cases^a^
	Significant summary associations (*p* < 10^−6^) per random‐effects calculations
	No evidence of small‐study effects
	No evidence of excess of significance bias
	Prediction intervals not including the null value
	Largest study nominally significant (*p* < .05)
	Not large heterogeneity (I^2^ < 50%)
Highly suggestive (class II)	>1000 cases^a^
	Significant summary associations (*p* < 10^−6^) per random‐effects calculations
	Largest study nominally significant (*p* < .05)
Suggestive (class III)	>1000 cases^a^
	Significant summary associations (*p* < 10^−3^) per random‐effects calculations
Weak (class IV)	Significant summary associations (*p* < .05) per random‐effects calculations
Nonsignificant association	Nonsignificant summary associations (*p* ≥ .05)

^a^ Total for the meta‐analysis.

The credibility of pooled estimates of meta‐analyses was qualitatively assessed using the GRADE (the Grading of Recommendations, Assessment, Development, and Evaluation) method.^(^ ^28^ ^)^ For each outcome, GRADE produces a credibility of estimate and summary of findings in a table that is easily understandable for study participants, policy makers, researchers, guideline developers, and other interested stakeholders (scoring detailed in Appendix Table AA2).^(^ ^28^ ^)^ AMSTAR‐2 and GRADE assessments were performed by one researcher (JDS) and verified by another (RS), and discrepancies were discussed and resolved by consensus.

**Table A2:** GRADE Assessment Scoring^a^

Study design	Quality of evidence	Lower if	Higher if
Randomized trial 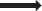	High	Risk of bias: ‐1 Serious ‐2 Very serious Inconsistency ‐1 Serious ‐2 Very serious Indirectness ‐1 Serious ‐2 Very serious Imprecision ‐1 Serious ‐2 Very serious Publication bias ‐1 Likely ‐2 Very likely	Large effect +1 Large +2 Very large Dose response +1 Evidence of a gradient All plausible confounding +1 Would reduce a demonstrated effect or +1 Would suggest a spurious effect when results show no effect
	Moderate		
Observational study 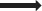	Low		
	Very low		

^a^ From: Guyatt G, Oxman AD, Akl EA, Kunz R, Vist G, Brozek J, et al. GRADE guidelines: 1. Introduction—GRADE evidence profiles and summary of findings tables. J Clin Epidemiol. 2011;64(4):383–94.

GRADE = Grading of Recommendations, Assessment, Development, and Evaluation.

## Results

### Meta‐analyses of observational studies

We found 35 meta‐analyses that investigated relationships between vitamin D status, as measured by circulating 25OHD and cancer outcomes: 29 on cancer incidence,^(^ ^5^, ^6^, ^11^, ^14^, ^29^, ^30^, ^31^, ^32^, ^33^, ^34^, ^35^, ^36^, ^37^, ^38^, ^39^, ^40^, ^41^, ^42^, ^43^, ^44^, ^45^, ^46^, ^47^, ^48^, ^49^, ^50^, ^51^, ^52^, ^53^ ^)^ 3 on cancer mortality,^10^, ^54^, ^55^ ^)^ and 3 on both^(^ ^13^, ^56^, ^57^ ^)^ (Table 1).

**Table 1:** Meta‐Analyses of Cohort Studies on the Association Between 25OHD and Cancer Outcomes

First author, publication year	Studies & design (*n*)	Participants (*n*)	Events (*n*)	Unit of 25OHD comparison	Pooled association (95% CI)	I^2^ (%) or *P* value for heterogeneity^a^
Cancer incidence						
All						
Han, 2019^(^ ^13^ ^)^	8 PC	70,018	7511	Highest vs. lowest group Per 20 nmol/L (dose–response)	RR, 0.86 (0.73–1.02) RR, 0.93 (0.91–0.96)	71 NR
Breast						
Chen, 2010^(^ ^29^ ^)^	7 (4 CC, 3 NCC)	11,330	5489	Highest vs. lowest quartile	OR, 0.55 (0.38–0.80)	86
Yin, 2010^(^ ^30^ ^)^	9 (5 CC, 4 NCC) 4 NCC	12,901 6327	6147 3117	Per 20 ng/mL	OR, 0.73 (0.60–0.88) OR, 0.92 (0.83–1.04)	84 NR
Chung, 2011^(^ ^31^ ^)^	4 NCC	4726	2363	Per 10 nmol/L (dose–response)	OR, 0.99 (0.97–1.01)	NR
Gandini, 2011^(^ ^32^ ^)^	10 (1 PC, 4 NCC, 5 CC) 5 (1 PC, 4, NCC)	29,742 23,078	6175 3145	Per 10 ng/mL	RR, 0.89 (0.81–0.98) RR, 0.97 (0.92–1.03)	88 54
Mohr, 2011^(^ ^33^ ^)^	11 (6 NCC, 5 CC) 6 NCC 5 CC	16,337 9673 6664	7547 4517 3030	Highest vs. lowest quintile Highest vs. lowest quintile Highest vs. lowest quintile	POR, 0.61 (0.47–0.80) POR, 0.87 (0.77–0.99) POR, 0.41 (0.31–0.56)	*p* < .0001 *p* = .50 *p* = .005
Bauer, 2013^(^ ^34^ ^)^	Premenopause: 6 PC Postmenopause: 9 PC	1613 3929	2890 8766	Per 5 ng/mL (dose–response)	RR, 0.99 (0.97–1.04) RR, 0.97 (0.93–1.00)	NR NR
Chen, 2013^(^ ^35^ ^)^	21 (10 NCC, 1 RSP, 10 CC) 11 NCC/RSP	26,317 6811	11771 15852	Highest vs. lowest quartile Highest vs. lowest quartile	OR, 0.52 (0.40–0.68) OR, 0.86 (0.75–1.00)	89 40
Wang, 2013^(^ ^49^ ^)^	14 (1 PC, 13 NCC) 11 (1 PC, 10 NCC)	25,354 20,252	9110 6715	Highest vs. lowest group Per 10 ng/mL (dose response)	RR, 0.84 (0.75–0.95) RR, 0.97 (0.94–0.99)	38 *p* = .13
Kim, 2014^(^ ^56^ ^)^	14 (1 PC, 13 NCC)	27,534	9526	Highest vs. lowest group Per 10 ng/mL (dose response)	RR, 0.92 (0.83–1.02) RR, 0.98 (0.96–1.00)	27 *P* value: NS
Estébanez, 2018^(^ ^50^ ^)^	29 (14 NCC, 15 CC)	58,855	18358	High vs. low group	OR, 0.66 (0.57–0.76)	41
	14 NCC	24,271	10266	High vs. low group	OR, 0.92 (0.83–1.01)	16
	4 PC	16,875	3350	Variable group comparisons	OR, 0.85 (0.74–0.98)	4
Hossain, 2019^(^ ^5^ ^)^	14 (12 NCC, 1 CC, 1 MR)	123,044	25515	Per 10 ng/mL	OR, 0.99 (0.98–1.00)	79
	5 CC	2796	1306	<10 ng/mL vs. ≥10 ng/mL	OR, 1.91 (1.51–2.41)	83
Song, 2019^(^ ^14^ ^)^	40 (4 PC, 36 CC)	162,322	31157	Per 5 nmol/L	OR, 0.94 (0.93–0.96)	91
Colon						
Yin, 2009^(^ ^36^ ^)^	6 NCC	2081	759	Per 20 ng/mL	OR, 0.78 (0.54–1.13)	45
Lee, 2011^(^ ^37^ ^)^	8 PC	4578	1822	Highest vs. lowest group	OR, 0.77 (0.56–1.07)	*p* = .04
Touvier, 2011^(^ ^6^ ^)^	6 NCC	3550	1477	Per 100 IU/L (dose–response)	RR, 0.95 (0.92–0.995)	48
Rectum						
Yin, 2009^(^ ^36^ ^)^	4 NCC	719	258	Per 20 ng/mL	OR, 0.41 (0.11–1.49)	63
Lee, 2011^(^ ^37^ ^)^	9 NCC	4578	868	Highest vs. lowest group	OR, 0.50 (0.28–0.88)	*p* = .04
Touvier, 2011^(^ ^6^ ^)^	5 NCC	1645	721	Per 100 IU/L	RR, 0.95 (0.89–1.05)	67
Colorectal						
Gorham, 2007^(^ ^38^ ^)^	5 NCC	1448	535	Highest vs. lowest group	POR, 0.49 (0.35–0.68)	*p* = .90
Yin, 2009^(^ ^36^ ^)^	5 NCC	3286	1199	Per 20 ng/mL	OR, 0.57 (0.43–0.76)	9
Chung, 2011^(^ ^31^ ^)^	9 NCC	2249	1127	Per 10 nmol/L	OR, 0.94 (0.91–0.97)	NR
Gandini, 2011^(^ ^32^ ^)^	9 (1 PC, 7 NCC, 1CC) 8 (1 PC, 7 NCC)	22,948 22,870	2630 2604	Per 10 ng/mL (dose–response) Per 10 ng/mL (dose–response)	RR, 0.85 (0.79–0.91) RR, 0.85 (0.79–0.92)	55 59
Lee, 2011^(^ ^37^ ^)^	8 NCC	4578	2690	Highest vs. lowest group	OR, 0.66 (0.54–0.81)	*p* = .04
Ma, 2011^(^ ^39^ ^)^	9 (7 PC, 2 NCC)	6715	2767	Highest vs. lowest group Per 10 ng/mL (dose–response)	RR, 0.67 (0.54–0.80) RR, 0.74 (0.63–0.89)	0 NR
Touvier, 2011^(^ ^6^ ^)^	6 NCC	5833	2370	Per 100 IU/L	RR, 0.96 (0.94–0.97)	0
Huang, 2019^(^ ^57^ ^)^	30 (6 PC, 23 NCC, 1 CC)	204,544	13051	Highest vs. lowest group	RR, 0.68 (0.60–0.78)	56
Zhang, 2019^(^ ^48^ ^)^	8 (1 NCC, 7 CC)	9594	2916	Highest vs. lowest group Per 16 ng/mL (dose–response)	OR, 0.75 (0.58–0.97) OR, 0.79 (0.64–0.97)	54 54
Colorectal adenoma						
Wei, 2008^(^ ^58^ ^)^	All adenomas: 7 (1 CS, 3 CC, 3 NCC/PC) Advanced adenomas: 2 NCC	3787 1023	2628 2347	Highest vs. lowest quintile High vs. low groups	OR, 0.70 (0.56–0.87) OR, 0.64 (0.45–0.90)	54 NR
Fedirko, 2010^(^ ^40^ ^)^	3 CC	1386	616	Highest vs. lowest quartile	OR, 0.59 (0.41–0.84)	NR
Yin, 2011^(^ ^41^ ^)^	Incident events: 9 (5 CC, 1 CS, 3 NCC) Recurrent events: 3 PC	7654 2169	3539 984	Per 20 ng/mL Per 20 ng/mL	OR, 0.82 (0.69–0.97) OR, 0.87 (0.56–1.35)	66 57
Huang, 2019^(^ ^57^ ^)^	22 (5 PC, 2 NCC, 14 CC, 1CS)	13652	6445	Highest vs. lowest group	RR, 0.80 (0.71–0.89)	34
Prostate						
Yin, 2009^(^ ^46^ ^)^	10 (1 PC, 9 NCC)	7806	3124	Per 10 ng/mL	OR, 1.03 (0.96–1.11)	23
Chung, 2011^(^ ^31^ ^)^	8 NCC	5609	2399	Per 10 nmol/L (dose–response)	OR, 1.01 (0.99–1.04)	NR
Gandini, 2011^(^ ^32^ ^)^	11 PC	26,575	3956	Per 10 ng/mL(dose–response)	RR, 0.99 (0.95–1.03)	37
Gilbert, 2011^(^ ^47^ ^)^	14 (5 PC, 9 NCC)	12,051	4353	Per 10 ng/mL	OR, 1.04 (0.99–1.10)	0
Kidney						
Gallicchio, 2010^(^ ^42^ ^)^	8 PC	1550	775	50‐ < 75 vs. ≥100 nmol/L	OR, 0.92 (0.44–1.92)	P‐value: NS
Liver						
Guo, 2020^(^ ^51^ ^)^	6 (1 PC, 5 NCC)	60,811	992	High vs. low group	RR, 0.78 (0.63–0.95)	54
				Per 10 nmol/L (dose–response)	RR, 0.92 (0.89–0.95)	NR
Lung						
Feng, 2017^(^ ^11^ ^)^	9 (6 PC, 3 CC)	111,148	1511	Variable group comparisons Per 10 nmol/L (dose–response)	RR, 0.84 (0.74–0.95) RR, 0.92 (0.87–0.96)	50 NR
Non‐Hodgkin lymphoma						
Purdue, 2010^(^ ^43^ ^)^	Males: 6 PC Females: 4 PC	923 923	733 733	>100 vs. 50–75 nmol/L >100 vs. 50–75 nmol/L	OR, 0.67 (0.37–1.20) OR, 0.81 (0.39–1.69)	NR NR
Ovarian						
Yin, 2011^(^ ^44^ ^)^	10 NCC	3373	884	Per 20 ng/mL	0.83 (0.63–1.08)	0
Pancreatic						
Stolzenberg‐Solomon, 2010^(^ ^45^ ^)^	6 NCC	833	345	≥100 vs. 50–75 nmol/L	OR, 2.14 (0.93–4.92)	*p* > .30
Thyroid						
Hu, 2018^(^ ^52^ ^)^	9 (7 CC, 1 CS, 1 RSP)	7099	1172	<20 vs. ≥20 ng/mL	OR, 1.42 (1.17–1.73)	27
	7 (5 CC, 2 CS)	6498	775	Cases vs. controls	SMD, –0.20 (−0.36 to −0.03)	55
Zhao, 2019^(^ ^53^ ^)^	6 CC	6241	711	Deficient vs. non‐deficient	OR, 1.30 (1.00–1.69)	38
	12 CC	7278	1239	Cases vs. controls	SMD, –0.37 (−0.45 to –0.28)	93
Cancer mortality						
All	Patients with					
Li, 2014^(^ ^54^ ^)^	breast cancer: 4 PC CRC: 3 (2 PC, 1 NC) lymphoma: 7 PC	4813 1558 1234	661 883 511	Highest vs. lowest quartile Highest vs. lowest quartile Highest vs. lowest quartile	HR, 0.65 (0.44–0.98) HR, 0.65 (0.47–0.88) HR, 0.50 (0.36–0.68)	45 6 0
Han, 2019^(^ ^13^ ^)^	16 PC	101,794	8729	Highest vs. lowest group Per 20 nmol/L (dose–response)	RR, 0.81 (0.71–0.93) RR, 0.98 (0.97–0.99)	49 NR
Breast						
Kim, 2014^(^ ^56^ ^)^	4 PC 3 PC	400 NR	4556 NR	Highest vs. lowest group Per 10 ng/mL (dose response)	RR, 0.58 (0.40–0.85) RR, 0.88 (0.79–0.98)	27 23
Maalmi, 2014^(^ ^10^ ^)^	3 PC	2636	194	High vs. low group	HR, 0.57 (0.38–0.84)	17
Colorectal						
Maalmi, 2014^(^ ^10^ ^)^	3 PC	1558	566	High vs. low group	HR, 0.65 (0.49–0.86)	0
Xu, 2018^(^ ^55^ ^)^	5 (3 PC, 2 NCC)	4126	982	High vs. low group	HR, 0.73 (0.55–0.97)	69
Huang, 2019^(^ ^57^ ^)^	12 PC	53,910	2021	High vs. low group	HR, 0.64 (0.56–0.73)	3

^a^ DerSimonian‐Laird Q statistic.

CC = Case control; CRC = colorectal cancer; CS = cross sectional; HR = hazard ratio; MR = Mendelian randomization; NCC = nested case–control; NR = not reported; NS = not significant; OR = odds ratio; PC = prospective cohort; POR = Peto odds ratio; RR = relative risk; RSP = retrospective; SMD = standardized mean difference.

#### Cancer incidence

With total cancer incidence as the outcome, one of these meta‐analyses combined data from eight prospective cohort studies (70,018 participants and 7511 events).^(^ ^13^ ^)^ The summary relative risk (RR) estimate of the highest 25OHD category compared with the lowest was 0.86 (95% CI, 0.73–1.02), indicating a marginal relationship in the inverse direction, with a significant between‐study heterogeneity (I^2^ = 70.8%). Dose–response analysis indicated a 7% reduction in risk (RR, 0.93; 95% CI, 0.91–0.96) per 20 nmol/L increment in 25OHD.

There were numerous meta‐analyses (43 from 32 articles) on site‐specific cancer outcomes: breast (*n* = 12), colon (*n* = 3), rectum (*n* = 3), colorectal (*n* = 9), colorectal adenomas (*n* = 4), prostate (*n* = 4), kidney (*n* = 1), liver (*n* = 1), lung (*n* = 1), non‐Hodgkin's lymphoma (*n* = 1), ovarian (*n* = 1), pancreatic (*n* = 1), and thyroid (*n* = 2). Most of these were prospective: cohort studies or case–control studies nested within these. The vast majority of associations were in the inverse direction. Of these, significant relationships were observed in most meta‐analyses of breast cancer (9 out of 12) and all meta‐analyses of colorectal cancer. Generally, heterogeneity was high for breast cancer (I^2^ up to 91%), but low to moderate for colorectal cancer (eg, I^2^ was 0% to 9% in three meta‐analyses). There were strong associations for some cancers: breast (lowest RR, 0.41^(^ ^33^ ^)^), colorectal (lowest RR, 0.49^(^ ^38^ ^)^), and rectum (lowest significant RR, 0.50^(^ ^37^ ^)^). Sizeable relationships were observed too for colorectal adenoma (lowest odds ratio [OR], 0.59^(^ ^40^ ^)^). Dose–response meta‐analyses reported inverse trends for breast,^(^ ^34^, ^49^, ^56^ ^)^ colon,^(^ ^6^ ^)^ colorectal,^(^ ^39^, ^48^ ^)^ liver,^(51^ ^)^ and lung^(^ ^11^ ^)^ cancers.

#### Cancer mortality

A recent meta‐analysis had total cancer mortality as the outcome.^(^ ^13^ ^)^ This report combined data from 16 prospective cohort studies on 101,794 participants without cancer at baseline, 8729 of whom had a cancer‐related death. The pooled RR—of a highest‐versus‐lowest 25OHD group comparison—was 0.81 (95% CI, 0.71–0.93), indicating an inverse association, with moderate heterogeneity (I^2^ = 48.8%). Dose–response analysis (of studies with ≥three categories of 25OHD) revealed that the risk of cancer mortality was reduced by 2% (RR, 0.98; 95% CI, 0.97–0.99) with each 20 nmol/L increment of 25OHD.

Four meta‐analysis articles of prospective studies focused on patients with cancer.^(^ ^10^, ^54^, ^55^, ^56^ ^)^ They reported sizeable inverse associations between 25OHD quantile (highest versus lowest) and cancer‐specific mortality: pooled hazard ratios (HRs) were 0.50 to 0.65 (in patients with breast cancer, colorectal cancer, and lymphoma) in one study,^(^ ^54^ ^)^ summary RR was 0.58 for breast cancer mortality in another study;^(^ ^56^ ^)^ the pooled RR ranged from 0.57 (for breast cancer mortality) to 0.65 (for colorectal cancer mortality) in another^(^ ^10^ ^)^; and the summary HR was 0.73 (for colorectal cancer mortality) in the fourth study.^(^ ^55^ ^)^ One of these studies reported an inverse dose–response association.^(^ ^56^ ^)^

### Meta‐analyses of randomized clinical trials

We identified eight meta‐analyses of clinical trials that evaluated the impact of vitamin D supplementation on cancer outcomes (incidence and mortality; Table 2). Appendix Table AA3 summarizes their study selection criteria, which excluded studies based on several factors such as coadministration of calcium that differed across the treatment groups,^(^ ^16^, ^19^ ^)^ treatment with hydroxylated vitamin D or vitamin D analogs,^(^ ^15^, ^17^, ^20^, ^21^ ^)^ duration of follow‐up (<1 year^(^ ^19^, ^20^ ^)^) or intervention (<3 years^(^ ^22^ ^)^, age (<18^(^ ^21^ ^)^ or <60^(^ ^19^ ^)^ years), number of outcomes (<10^(^ ^20^ ^)^), and pregnancy.^(^ ^15^, ^16^, ^21^ ^)^

**Table 2:** Meta‐Analyses of Intervention Studies^a^ on the Effect of Vitamin D Supplementation on Cancer Outcomes

Study	Studies (*n*)	Sample size (*n*)	Events (*n*)	Pooled RR effect (95% CI)	NNT (95% CI)	I^2^ (%)	Quality of meta‐analysis (AMSTAR‐2 rating)	Strength of association (umbrella review class)	GRADE credibility of evidence
Cancer incidence									
All									
Bolland, 2014^(^ ^17^ ^)^	7	48,167	3979	0.99 (0.93–1.05)	—	0	Critically low	NS	Moderate
Bjelakovic, 2014^(^ ^16^ ^)^	14	49,891	3851	1.00 (0.94–1.06)	—	0	High	NS	Moderate
Keum, 2014^(^ ^18^ ^)^	4	45,151	4333	1.00 (0.94–1.06)	—	0	Critically low	NS	High
Goulão, 2018^(^ ^19^ ^)^	24	18,440	1061	1.03 (0.91–1.15)	—	0	Critically low	NS	High
Haykal, 2019^(^ ^22^ ^)^	9	42,773	3022	0.96 (0.86–1.07)	—	31	Critically low	NS	High
Keum, 2019^(^ ^20^ ^)^	10	83,353	6537	0.98 (0.93–1.03)	—	0	Critically low	NS	High
Breast									
Sperati, 2013^(^ ^15^ ^)^	2	5372	91	1.11 (0.74–1.68)	—	0	Moderate	NS	Low
Bjelakovic, 2014^(^ ^16^ ^)^	7	43,669	1135	0.97 (0.86–1.09)	—	0	High	NS	Moderate
Colorectal									
Bjelakovic, 2014^(^ ^16^ ^)^	5	45,598	436	1.11 (0.92–1.34)	—	0	High	NS	Moderate
Lung									
Bjelakovic, 2014^(^ ^16^ ^)^	5	45,509	329	0.86 (0.69–1.07)	—	0	High	NS	Moderate
Pancreatic									
Bjelakovic, 2014^(^ ^16^ ^)^	2	36,405	69	0.91 (0.57–1.46)	—	0	High	NS	Moderate
Cancer mortality									
All									
Bjelakovic, 2014^(^ ^16^ ^)^	4	44,492	1192	0.88 (0.78–0.98)	292 (159–1751)	0	High	Weak	High
Keum, 2014^(^ ^18^ ^)^	3	44,290	1190	0.88 (0.78–0.98)	86 (47–515)	0	Critically low	Weak	High
Goulão, 2018^(^ ^19^ ^)^	7	11,202	320	0.88 (0.70–1.09)	—	0	Critically low	NS	Moderate
Haykal, 2019^(^ ^22^ ^)^	5	70,547	1533	0.87 (0.79–0.96)	381 (236–1238)	0	Low	Weak	High
Keum, 2019^(^ ^20^ ^)^	5	75,239	1591	0.87 (0.79–0.96)	294 (182–957)	0	Critically low	Weak	High
Zhang, 2019^(^ ^21^ ^)^	12	45,578	939	0.84 (0.74–0.95)	279 (171–892)	0	Moderate	Weak	High

^a^ All were randomized controlled trials.

AMSTAR = A Measurement Tool to Assess Systematic Reviews; GRADE = Grading of Recommendations, Assessment, Development, and Evaluation; NNT = number needed to treat; NS = nonsignificant association; RR = relative risk.

**Table A3:** Study Selection Criteria of Meta‐Analyses of Intervention Studies^a^ on the Effect of Vitamin D Supplementation on Cancer Outcomes

Meta‐analysis	Inclusion criteria	Exclusion criteria
Sperati, 2013^(^ ^15^ ^)^	1. Compared with placebo/no treatment 2. Vitamin D as single agent 3. Combined regimens including supplements & lifestyle modifications if used equally in all groups	Pregnant or lactating women
Bjelakovic, 2014^(^ ^16^ ^)^	1. RCTs, irrespective of blinding, publication, status, or language. 2. Any dose, duration, and route of administration 3. Monotherapy or in combination with calcium 4. Concomitant interventions if used equally in all intervention groups	1. Secondary induced osteoporosis (eg, glucocorticoid‐induced osteoporosis, thyroidectomy, primary hyperparathyroidism, chronic kidney disease, liver cirrhosis, Crohn disease, gastrointestinal bypass surgery) 2. Pregnant or lactating women 3. People with cancer
Bolland, 2014^(^ ^17^ ^)^	Cholecalciferol or ergocalciferol	1. Cluster randomized trials 2. Trials of hydroxylated vitamin D or vitamin D analogues 3. Other interventions only in vitamin D group 4. Trials of fortified dairy products 5. Chronic comorbidity other than osteoporosis or frailty
Keum, 2014^(^ ^18^ ^)^	With or without calcium supplementation	1. Non‐English articles 2. Abstracts & unpublished reports
Goulão, 2018^(^ ^19^ ^)^	1. Mean or median age of ≥60 years 2. Follow‐up ≤1 year 3. Any vitamin D or vitamin D analog 4. Coadministration of other medications (eg, calcium) if the comparator group received the same medication 5. All languages	1. Renal impairment, steroid‐induced osteoporosis, or psoriasis 2. Nonmelanoma skin cancers not counted as events
Haykal, 2019^(^ ^22^ ^)^	1. Primary prevention 2. Vitamin D compared with placebo 3. Vitamin D for ≥3 years	
Keum, 2019^(^ ^20^ ^)^	Cholecalciferol or ergocalciferol, with or without other nutrients	1. Number of outcomes ≤10 2. Follow‐up ≤1 year
Zhang, 2019^(^ ^21^ ^)^	1. Age ≥ 18 years 2. Any health conditions 3. Vitamin D (any dose) vs. placebo or no treatment 4. Concomitant agents had to be same dose in all groups	1. Case reports, case series, observational studies 2. All participants received vitamin D 3. Pregnant or lactating women 4. Critically patients 5. Hydroxylated vitamin D or vitamin D analogues

^a^ All were randomized controlled trials (RCTs).

#### Quality of meta‐analyses

The AMSTAR‐2 ratings of the eight meta‐analyses are summarized in Table 2 and detailed in Appendix Table AA4. Five meta‐analyses had shortcomings in critical domains^(^ ^17^, ^18^, ^19^, ^20^, ^22^ ^)^: All five did not cite a predefined, registered protocol, four did not cite excluded primary studies,^(^ ^18^, ^19^, ^20^, ^22^ ^)^ three did not use a satisfactory technique to assess risk of bias^(^ ^17^, ^18^, ^20^ ^)^, and two used a literature search that was not fully comprehensive.^(^ ^17^, ^18^ ^)^ All studies had deficiencies in noncritical domains, with the most common being reporting funding sources of primary studies (absent in seven meta‐analyses^(^ ^15^, ^17^, ^18^, ^19^, ^20^, ^21^, ^22^ ^)^) and explaining selection of study designs for inclusion in the review (absent in six meta‐analyses^(^ ^15^, ^16^, ^17^, ^19^, ^21^, ^22^ ^)^). Based on weaknesses in the critical and noncritical domains, the overall confidence in the eight meta‐analyses was deemed critically low or moderate for seven of these.^(^ ^15^, ^17^, ^18^, ^19^, ^20^, ^21^, ^22^ ^)^

**Table A4:** AMSTAR‐2 Ratings of Meta‐Analyses of Intervention Studies on the Effect of Vitamin D Supplementation on Cancer Outcomes

AMSTAR‐2 item		First author, publication year (citation)							
Item	Description	Bolland, 2014^(^ ^17^ ^)^	Sperati, 2013^(^ ^15^ ^)^	Bjelakovic, 2014^(^ ^16^ ^)^	Keum, 2014^(^ ^18^ ^)^	Goulão, 2018^(^ ^19^ ^)^	Haykal, 2019^(^ ^22^ ^)^	Keum, 2019^(^ ^20^ ^)^	Zhang, 2019^(^ ^21^ ^)^
1	Did the research questions and inclusion criteria include the components of PICO?	✓	✓	✓	✓	✓	✓	✓	✓
2^a^	Did the review contain an explicit statement that the review methods were established prior to the conduct of the review and did the report justify any significant deviations from the protocol?	×	✓	✓	×	×	×	×	✓
3	Did the review authors explain their selection of the study designs for inclusion in the review?	×	×	×	✓	×	×	✓	×
4^a^	Did the review authors use a comprehensive literature search strategy?	×	✓	✓	×	✓	Partial ✓	Partial ✓	✓
5	Did the review authors perform study selection in duplicate?	×	✓	✓	✓	×	✓	✓	✓
6	Did the review authors perform data extraction in duplicate?	✓	✓	✓	✓	✓	✓	✓	✓
7^a^	Did the review authors provide a list of excluded studies and justify the exclusions?	✓	✓	✓	×	×	×	×	✓
8	Did the review authors describe the included studies in adequate detail?	✓	✓	✓	✓	✓	✓	✓	Partial ✓
9^a^	Did the review authors use a satisfactory technique to assess the RoB in studies that were included in the review?	×	✓	✓	×	✓	✓	×	✓
10	Did the review authors report on the sources of funding for the studies included in the review?	×	×	✓	×	×	×	×	×
11^a^	If meta‐analysis was performed did the review authors use appropriate methods for statistical combination of results?	✓	✓	✓	✓	✓	✓	✓	✓
12	If meta‐analysis was performed, did the review authors assess the potential impact of RoB in individual studies on the results of the meta‐analysis or other evidence synthesis?	×	✓	✓	×	✓	✓	✓	✓
13^a^	Did the review authors account for RoB in individual studies when interpreting/discussing the results of the review?	×	✓	✓	×	✓	✓	×	✓
14	Did the review authors provide a satisfactory explanation for, and discussion of, any heterogeneity observed in the results of the review?	✓	✓	✓	✓	✓	✓	✓	✓
15	If they performed quantitative synthesis did the review authors investigate publication bias and discuss its likely impact on the results?	✓	✓	✓	✓	✓	✓	✓	✓
16	Did the review authors report any potential sources of conflict of interest, including any funding they received for conducting the review?	✓	✓	✓	✓	✓	✓	✓	✓
	Rating of overall confidence in the results of the review	CL	Moderate	High	CL	CL	CL	CL	Moderate

^a^ Critical domains.

AMSTAR = A Measurement Tool to Assess Systematic Reviews; CL = critically low; PICO components: P = patient population/problem, I = intervention (issue of interest, considered for implementation), C = comparison (comparitor or current practice), O = outcome (how this is measured); RoB = risk of bias.

#### Cancer incidence

Six of these meta‐analyses had total cancer incidence as an outcome.^(^ ^16^, ^17^, ^18^, ^19^, ^20^, ^22^ ^)^ These reports were published between 2014 and 2019, with each comprising 4 to 18 studies, 18,440 to 83,353 participants, and 1061 to 6537 cancer incident events. The pooled RR effects in these reports were similar, ranging from 0.98 (10 studies)^(^ ^20^ ^)^ to 1.03 (24 studies),^(^ ^19^ ^)^ with all 95% CIs encompassing 1. The I^2^ for these effects was 0%^(^ ^16^, ^17^, ^18^, ^19^, ^20^ ^)^ or 31%,^(^ ^22^ ^)^ indicating no significant heterogeneity.

Two of these meta‐analyses had specific cancer incidence as an outcome.^(^ ^15^, ^16^ ^)^ In these, the pooled RR effects were 0.97 (seven studies) to 1.11 (two studies) for breast cancer, 0.86 (95% CI, 0.69–1.07) for lung cancer (five studies), 1.11 (95% CI, 0.92–1.34) for colorectal cancer, and 0.91 (95% CI, 0.57–1.46) for pancreatic cancer (two studies).

#### Cancer mortality

Six of these meta‐analyses had cancer mortality as an outcome.^(^ ^16^, ^18^, ^19^, ^20^, ^21^, ^22^ ^)^ The pooled RR effects in these reports were similar and all in the inverse direction, ranging from 0.84 (95% CI, 0.74–0.95; 12 studies) to 0.88 (95% CI, 0.70–1.09; 24 studies). The 95% CIs did not encompass one in five out of six of these meta‐analyses,^(^ ^16^, ^18^, ^20^, ^21^, ^22^ ^)^ indicating consistently beneficial impacts on cancer mortality.

#### Strength and credibility of meta‐analysis effects

Five intervention meta‐analyses of total cancer mortality had weak strength of association according to umbrella review criteria,^(^ ^16^, ^18^, ^20^, ^21^, ^22^ ^)^ with all scoring high with GRADE and with NNT values ranging from 86 to 381. This was consistent among meta‐analyses of high, moderate, and lower quality (AMSTAR‐2–rated). The remaining meta‐analyses reported nonsignificant effects, with GRADE credibility that was moderate^(^ ^16^, ^17^ ^)^ to high^(^ ^18^, ^19^, ^20^, ^22^ ^)^ for total cancer incidence and low^(^ ^15^, ^16^ ^)^ to moderate^(^ ^16^ ^)^ for site‐specific cancer incidence. A breakdown of these scores is provided in Appendix Tables AA5 and AA6.

**Table A5:** Umbrella Review Assessment of Meta‐Analyses of Intervention Studies on the Effect of Vitamin D Supplementation on Cancer Outcomes

Reference	RR Effect (95% CI)	>1000 events (cases)	Significant summary associations per random‐effects calculations			No evidence of small‐study effects	No evidence of excess of significance	Prediction intervals excluding null value	Largest study nominally significant (*p* < .05)	Not large heterogeneity (I^2^ <50%)	Umbrella review class
			*p* < 10^−6^	<10^−3^	*p* < .05						
All cancers											
Bolland, 2014^(^ ^17^ ^)^	0.99 (0.93–1.05)	✓	×	×	×	✓	✓	×	×	✓	NS association
Bjelakovic, 2014^(^ ^16^ ^)^	1.00 (0.94–1.06)	✓	×	×	×	×	✓	×	×	✓	NS association
Keum, 2014^(^ ^18^ ^)^	1.00 (0.94–1.06)	✓	×	×	×	✓	✓	×	×	✓	NS association
Goulão, 2018^(^ ^19^ ^)^	1.03 (0.91–1.15)	✓	×	×	×	✓	✓	×	×	✓	NS association
Haykal, 2019^(^ ^22^ ^)^	0.96 (0.86–1.07)	✓	×	×	×	✓	✓	×	×	✓	NS association
Keum, 2019^(^ ^20^ ^)^	0.98 (0.93–1.03)	✓	×	×	×	✓	✓	×	×	✓	NS association
Breast cancer											
Sperati, 2013^(^ ^15^ ^)^	1.11 (0.74–1.68)	×	×	×	×	✓	✓	×	×	✓	NS association
Bjelakovic, 2014^(^ ^16^ ^)^	0.97 (0.86–1.09)	✓	×	×	×	✓	✓	×	×	✓	NS association
Colorectal cancer											
Bjelakovic, 2014^(^ ^16^ ^)^	1.11 (0.92–1.34)	×	×	×	×	✓	✓	×	×	✓	NS association
Lung cancer											
Bjelakovic, 2014^(^ ^16^ ^)^	0.86 (0.69–1.07)	×	×	×	×	✓	✓	×	×	✓	NS association
Pancreatic cancer											
Bjelakovic, 2014^(^ ^16^ ^)^	0.91 (0.57–1.46)	×	×	×	×	✓	✓	×	×	✓	NS association
Total cancer mortality											
Bjelakovic, 2014^(^ ^16^ ^)^	0.88 (0.78–0.98)	✓	×	×	✓	×	✓	✓	×	✓	Weak (class IV)
Keum, 2014^(^ ^18^ ^)^	0.88 (0.78–0.98)	✓	×	×	✓	✓	✓	✓	×	✓	Weak (class IV)
Goulão, 2018^(^ ^19^ ^)^	0.88 (0.70–1.09)	×	×	×	×	✓	✓	×	×	✓	NS association
Haykal, 2019^(^ ^22^ ^)^	0.87 (0.79–1.06)	×	×	×	✓	✓	✓	✓	×	✓	Weak (class IV)
Keum, 2019^(^ ^20^ ^)^	0.87 (0.79–0.96)	✓	×	×	✓	✓	✓	✓	×	✓	Weak (class IV)
Zhang, 2019^(^ ^21^ ^)^	0.84 (0.74–0.95)	×	×	×	✓	✓	✓	✓	×	✓	Weak (class IV)

NS = nonsignificant; RCT = randomized controlled trial; RR = relative risk.

**Table A6:** GRADE Summary of Findings for Meta‐Analyses of Intervention Studies on the Effect of Vitamin D Supplementation on Cancer Outcomes

Reference	Studies (*N*)	Study design	Risk of bias	Imprecision	Inconsistency	Indirectness	Publication bias	RR Effect (95% CI)	Certainty (GRADE)
All cancers									
Bolland, 2014^(^ ^17^ ^)^	7	RCT	Not serious	Not serious	Not serious	Not serious	Possible: Egger's *P* value = 0.05	0.99 (0.93–1.05)	+++ Moderate
Bjelakovic, 2014^(^ ^16^ ^)^	14	RCT	Not serious	Not serious	Not serious	Not serious	Likely: Egger's *P* value = 0.007	1.00 (0.94–1.06)	+++ Moderate
Keum, 2014^(^ ^18^ ^)^	4	RCT	Not serious	Not serious	Not serious	Not serious	Unlikely	1.00 (0.94–1.06)	++++ High
Goulão, 2018^(^ ^19^ ^)^	24	RCT	Not serious	Not serious	Not serious	Not serious	Unlikely	1.03 (0.91–1.15)	++++ High
Haykal, 2019^(^ ^22^ ^)^	9	RCT	Not serious	Not serious	Not serious	Not serious	Unlikely	0.96 (0.86–1.07)	++++ High
Keum, 2019^(^ ^20^ ^)^	10	RCT	Not serious	Not serious	Not serious	Not serious	Unlikely	0.98 (0.93–1.03)	++++ High
Breast cancer									
Sperati, 2013^(^ ^15^ ^)^	2	RCT	Not serious	Serious: wide CI from benefit to appreciable harm	Not serious	Not serious	Unlikely	1.11 (0.74–1.68)	++ Low
Bjelakovic, 2014^(^ ^16^ ^)^	7	RCT	Not serious	Not serious	Not serious	Not serious	Unlikely	0.97 (0.86–1.09)	+++ Moderate
Lung cancer									
Bjelakovic, 2014^(^ ^16^ ^)^	5	RCT	Not serious	Not serious	Not serious	Not serious	Unlikely	0.86 (0.69–1.07)	+++ Moderate
Colorectal cancer									
Bjelakovic, 2014^(^ ^16^ ^)^	5	RCT	Not serious	Not serious	Not serious	Not serious	Unlikely	1.11 (0.92–1.34)	+++ Moderate
Pancreatic cancer									
Bjelakovic, 2014^(^ ^16^ ^)^	2	RCT	Not serious	Serious: wide CI from appreciable benefit to appreciable harm	Not serious	Not serious	Too few studies to assess	0.91 (0.57–1.46)	+++ Moderate
Total cancer mortality									
Bjelakovic, 2014^(^ ^16^ ^)^	4	RCT	Not serious	Not serious	Not serious	Not serious	Unlikely	0.88 (0.78–0.98)	++++ High
Keum, 2014^(^ ^18^ ^)^	3	RCT	Not serious	Not serious	Not serious	Not serious	Unlikely	0.88 (0.78–0.98)	++++ High
Goulão, 2018^(^ ^19^ ^)^	7	RCT	Not serious	Serious: wide CI from appreciable benefit to small harm	Not serious	Not serious	Unlikely	0.88 (0.70–1.09)	+++ Moderate
Haykal, 2019 (22)	5	RCT	Not serious	Not serious	Not serious	Not serious	Unlikely	0.87 (0.79–0.96)	++++ High
Keum, 2019^(^ ^20^ ^)^	5	RCT	Not serious	Not serious	Not serious	Not serious	Unlikely	0.87 (0.79–0.96)	++++ High
Zhang, 2019^(^ ^21^ ^)^	12	RCT	Not serious	Not serious	Not serious	Not serious	Unlikely	0.84 (0.74–0.95)	++++ High

RCT = randomized controlled trial; RR = relative risk.

### Differences between trial meta‐analyses

As pooled effects across meta‐analyses on the same outcome were mostly similar (with overlapping 95% CIs; Table 2) despite variable study selection criteria (Appendix Table AA3), this enhances the external validity of our findings that vitamin D reduces total cancer mortality (in nearly all meta‐analyses), but not cancer incidence. Where pooled effects for the same outcome variations did vary, this can be explained by differences in the number of primary studies in meta‐analyses, which is influenced by publication year (Table 2) and study selection criteria. The most important variation occurred for cancer mortality, in which the meta‐analysis by Goulão and colleagues^(^ ^19^ ^)^ did not find a significant overall effect, but the remaining five meta‐analyses did (Table 2). Goulão and colleagues^(^ ^19^ ^)^ excluded primary studies in which calcium was coadministered with vitamin D, but not with placebo, which allowed it to disentangle calcium and vitamin D effects. This restriction meant that the Women's Health Initiative,^(^ ^59^ ^)^ in which calcium was given with vitamin D in the intervention group only, was not included in this meta‐analysis (unlike nearly all other ones^(^ ^16^, ^18^, ^20^, ^22^ ^)^). However, this exclusion may not have been important for validity if calcium did not influence cancer mortality, as suggested by prior studies.^(^ ^60^ ^)^ Also, the number of participants (*n* = 11202) and events (*n* = 320) in the Goulão and colleagues^(^ ^19^ ^)^ meta‐analysis was substantially lower than those in the other meta‐analyses (*n* = 44,290 to 75,239 and *n* = 939 to 1192 for number of participants and events, respectively; Table 2). This is because two large (*n* > 5000) trials in addition to the Women's Health Initiative^(59)^—the VIDA (Vitamin D Assessment)^(^ ^61^ ^)^ and VITAL (Vitamin D and Omega‐3)^(^ ^62^ ^)^ studies—were included in other meta‐analyses (showing vitamin D benefits),^20^, ^21^, ^22^ ^)^ but not in the meta‐analysis by Goulão and colleagues^(^ ^19^ ^)^ as they were published after it.^(^ ^19^ ^)^ Thus, the Goulão and colleagues’^(^ ^19^ ^)^ meta‐analysis did not capture effects from some large trials and had reduced statistical power.

### Limitations of meta‐analyses and their primary studies

Owing to several limitations, the meta‐analyses and their primary studies have boundaries of applicability. These limitations influence the strength of associations and are key targets for future research. We discuss these for observational and intervention studies separately.

#### Observational studies

A limitation of the observational studies we reviewed is that, although they adjusted for multiple confounders, they are vulnerable to residual confounding; more so when the confounders (eg, smoking behavior, BMI, physical activity, diet) are measured less well. Adding to this issue is that vitamin D status is related to multiple diseases, besides cancer.^(^ ^63^ ^)^ Thus, 25OHD–cancer associations do not fulfill one of Bradford‐Hill's causation criteria, specificity,^(^ ^64^ ^)^ raising the possibility that confounding may be important. On the other hand, confounding may not entirely explain these associations as risk factors can cause more than one disease,^(64^ ^)^ and vitamin D has pleiotropic cellular effects.^(^ ^63^ ^)^ A second limitation is that several observational studies, especially those on breast cancer incidence,^(^ ^14^, ^29^, ^30^, ^32^, ^33^, ^35^ ^)^ were case–control investigations—and are thus susceptible to reverse causation because 25OHD measurement was performed in those already diagnosed with cancer, and low vitamin D status may be a consequence of the disease rather than a cause. For instance, during cancer therapy or when symptoms are severe, sunlight exposure, physical activity, and dietary habits are likely to change (because of hospitalizations, disability, or lifestyle changes), and cancer‐associated inflammation may depress 25OHD.^(^ ^65^ ^)^ However, this problem was avoided in prospective studies where blood samples were collected well before cancer diagnosis. The studies in the meta‐analyses we reviewed were mainly prospective (nested case–control or cohort) and we observed inverse associations in them, suggesting that 25OHD may affect cancer (Table 1) rather than vice versa. Third, several observational studies are prone to 25OHD measurement error because they did not measure vitamin D status with the gold standard laboratory method for 25OHD measurement: liquid chromatography–tandem mass spectrometry.^(^ ^66^ ^)^ Fourth, whereas many observational studies were of high quality, as assessed by the Newcastle‐Ottawa Scale, several were of suboptimal (medium) quality, which may have attenuated 25OHD–cancer associations. In support of this, a recent meta‐analysis reported that 25OHD was inversely associated with total cancer incidence and mortality in studies of high quality, but not in those of medium quality.^(^ ^13^ ^)^ Fifth, though most meta‐analyses had low or moderate between‐study heterogeneity (I^2^ < 50% or *P* values >0.05),^(^ ^6^, ^31^, ^36^, ^38^, ^39^, ^42^, ^44^, ^45^, ^47^, ^49^, ^50^, ^52^, ^53^, ^54^, ^56^, ^57^ ^)^ some did not; thus, their results should be interpreted with caution. These include meta‐analyses on colorectal adenoma and cancer occurrences of the breast, rectum, and all types combined,^5^, ^6^, ^13^, ^14^, ^29^, ^30^, ^32^, ^35^, ^36^ ^)^ which reported high between‐study heterogeneity (I^2^ ≥ 50%). Finally, most participants in the observational studies were of White ethnicity, which limits generalizability of findings to non‐White ethnic groups.

#### Randomized clinical trials

An important issue for intervention studies is the growing randomized controlled trial (RCT) evidence that health benefits of vitamin D supplementation are greatest in vitamin D‐deficient people.^(^ ^67^ ^)^ This may also apply to cancer as a recent meta‐analysis of cohort studies revealed that 25OHD was inversely associated with cancer incidence at approximately <30 nmol/L only, and had an inverse relationship with cancer mortality that was strongest at low 25OHD levels (especially at approximately <50 nmol/L).^(^ ^13^ ^)^ Thus, any cancer‐related benefits of vitamin D supplementation may be greatest in vitamin D‐deficient individuals. However, trials have not contained large numbers of such people. For example, the average baseline 25OHD of 10 trials examined in the recent meta‐analysis by Keum and colleagues^(^ ^20^ ^)^ we reviewed was approximately 60 nmol/L.

Insufficient vitamin D dosage may be another limitation. For example, in the Goulão and colleagues meta‐analysis of cancer incidence,^(^ ^19^ ^)^ the daily dose equivalent (dose divided by days between each dose) in multiple primary studies (*n* = 7) was relatively low (<1000 IU/day), raising a question of whether a higher dose may have produced a different (stronger) effect. A related issue is the frequency of the dosing regimens. Most trials have utilized daily dosing; evidence is limited for supplementation administered monthly or weekly.^(^ ^20^ ^)^

Third, as carcinogenesis is a long‐term and gradual process (often spanning decades), the need for a long follow‐up period is particularly great.^(^ ^68^ ^)^ The importance of this is reflected in the long follow‐up periods of 25OHD–cancer cohort studies, such as those in the Han and colleagues’ meta‐analysis (of total cancer incidence and mortality),^(^ ^13^ ^)^ which were 12 to 13 years on average and up to 28 years. However, most of the primary studies included in our meta‐analyses of trials had follow‐up periods of no more than 5 years; this may have been insufficient to detect effects on cancer.^(^ ^68^ ^)^ In support of this, the meta‐analysis by Zhang and colleagues^(^ ^21^ ^)^ we reviewed found that benefit of supplementation on reduced cancer mortality was observed in trials with longer follow‐up (>3 years), but not in those with a shorter follow‐up.

A fourth limitation is that, as shown in the meta‐analyses for observational studies, 25OHD was more consistently associated with colorectal and breast cancers than other cancers (Table 1). Thus, there may be stronger effects of supplementation against certain cancer types or, possibly, residual confounding may be greater for some cancers than others (eg, confounding by BMI, physical activity, and diet may be of particular relevance to studies of vitamin D and colorectal cancer). However, whereas the cancer outcomes that have dominated our RCT findings are those based on all cancers combined (Table 2), results for site‐specific cancers are lacking. Almost all trials that evaluated impacts on site‐specific cancers did not include these as primary endpoints, and data on rarer cancers (eg, kidney) are missing.

Fifth, the vast majority of participants in the intervention studies were White, which restricts applicability of findings to non‐White populations.

Finally, there were quality‐related shortcomings of the meta‐analyses of intervention studies we reviewed. The most common ones, detected by AMSTAR‐2, were the lack of (i) information on funding sources of primary studies (noteworthy as many vitamin D trials are industry‐funded and have a high risk of “for‐profit” bias^(^ ^16^ ^)^), (ii) an explanation of study design selection, and (iii) a review protocol describing prespecified methodology. Most of these meta‐analyses were published prior to the availability of AMSTAR‐2 reporting standards (in 2017^(^ ^9^ ^)^), which may have contributed to their lower AMSTAR‐2 ratings. However, as mentioned above, our vitamin D–cancer findings for meta‐analyses of high and moderate quality (AMSTAR‐2–rated) were similar to those of lower quality.

### Limitations of current review

A potential limitation of this review is that, although a comprehensive and systematic literature search was performed, we may have missed some meta‐analyses. Second, our study was a meta‐review, and although this provides an overarching perspective on a research topic, we did not provide granulate analyses at the primary study level. Third, our review focused on meta‐analyses; thus, some primary studies may not have been included either because the meta‐analysis did not identify them or they were too recent to be included. Finally, we did not critically appraise the quality of all primary studies individually. This should have been done in each meta‐analysis; doing this here was beyond the scope of our review.

### Future research

Several areas of future research would strengthen our understanding of vitamin D effects on cancer. First, given the emerging evidence for threshold effects related to vitamin D status, future trials should aim to recruit participants with vitamin D insufficiency (25OHD <50 nmol/L). There are major logistical and practical barriers to doing this in populations that are vitamin D replete, and trials could be undertaken more easily and cheaply in populations with a high prevalence of vitamin D insufficiency. These trials should have longer follow‐up periods, include more adequately powered data on site‐specific cancers (feasible for common cancers), and study more non‐White populations. However, ethical issues can arise with the conduct of long‐term trials in vitamin D‐deficient participants, as 50% will be randomly assigned to placebo and remain deficient for a prolonged period.

Second, cells that express the cell‐surface receptor proteins megalin and cubulin (eg, those in the kidney, lung, thyroid, mammary gland, gall bladder, and thyroid) can internalize 25OHD bound to vitamin D‐binding protein, with subsequent unbinding of 25OHD intracellularly and conversion to 1,25‐dihydroxyvitamin D, which can exert anticancer effects by activating the vitamin D receptor.^(^ ^69^, ^70^ ^)^ In contrast, 25OHD entry in cells not expressing the megalin–cubulin receptor is proposed to occur via diffusion of unbound, free 25OHD across the cell membrane.^(^ ^69^, ^70^ ^)^ However, the studies we reviewed measured total 25OHD, comprising not only free (and bioavailable) 25OHD, but mostly (approximately 90%) 25OHD, which is bound tightly to vitamin D‐binding protein, and thus may not get into some cancer cells easily.^(^ ^71^ ^)^ As evidence of importance, a large, prospective cohort study reported that higher bioavailable, rather than total, 25OHD levels were independently associated with improved survival in patients with hepatocellular carcinoma.^(^ ^72^ ^)^ Further, in a recent RCT of patients with digestive tract cancer, vitamin D supplementation improved 5‐year relapse‐free survival in those with low bioavailable 25OHD, but not in those with high bioavailable 25OHD^(^ ^71^ ^)^ or in those with low total 25OHD (<20 ng/mL).^(^ ^73^ ^)^ These studies suggest that for some cancer types, free and bioavailable 25OHD may better assess true vitamin D status (and deficiency) than total 25OHD in future trials.

Third, articles that reported inverse, longitudinal associations between 25OHD and cancer mortality^(^ ^10^, ^54^, ^55^, ^56^ ^)^ (Table 1) were studies of patients with cancer, and thus suggested a potential role of vitamin D in cancer therapy as opposed to cancer prevention, the focus of the other articles we reviewed. RCT data investigating vitamin D as a cancer treatment are scarce, with one relatively small RCT (*n* = 139) reporting an improvement in median progression‐free survival or death (HR, 0.64) over 22.9 months (median) in patients with colorectal cancer^(74^ ^)^ and another (*n* = 417) reporting an age‐adjusted benefit on relapse‐free survival (HR, 0.66) over 3.5 years (median) in patients with digestive tract cancers.^(^ ^73^ ^)^ However, further trials are required in this research area, ideally with longer follow‐up.^(^ ^75^ ^)^ Including biological measurements would promote our understanding of underlying mechanisms: This requires considering not only antineoplastic influences, but broad biological effects too, as 25OHD is inversely related to all‐cause mortality in patients with or without cancer.^(^ ^75^ ^)^

Fourth, cancer incidence and mortality, and overall survival, though considered the gold standard endpoint in oncology trials, require a large sample size and long follow‐up time to achieve adequate statistical power.^(^ ^76^ ^)^ In comparison, other clinical endpoints can be assessed earlier, and thus could be measured in parallel. One of these is health‐related quality of life, which is considered an outcome in assessing clinical benefit and has emerged as a primary endpoint in oncology clinical trials.^(^ ^76^ ^)^ Encouragingly, observational cohort studies found that, in patients with cancer, vitamin D intake and 25OHD predict improved quality of life,^(^ ^77^, ^78^, ^79^ ^)^ but RCT data are needed to evaluate effects on this outcome. Another is tumor‐centered endpoints, such as progression‐free survival, disease‐free survival, tumor response, and circulating tumor cells.^(^ ^76^ ^)^ As mentioned, two RCTs reported beneficial effects on median progression‐free survival^(^ ^74^ ^)^ and relapse‐free survival,^(^ ^73^ ^)^ but further RCTs are needed.^(^ ^75^ ^)^ Thus, adding these other clinical endpoints (alongside gold standard outcomes) to future vitamin D trials would provide an earlier assessment and more comprehensive evaluation of efficacy.^(^ ^76^ ^)^

Finally, a novel area of research is investigating whether vitamin D pathway genes may alter health effects on vitamin D supplementation. A meta‐analysis of eight prospective studies found that colorectal cancer risk was lower in participants with the BB genotype of the *BsmI* vitamin D receptor single‐nucleotide polymorphism.^(^ ^6^ ^)^ A recent RCT found that vitamin D receptor genotypes modified the effect of vitamin D supplementation on the prevention of advanced colorectal adenomas.^(^ ^80^ ^)^ Specifically, vitamin D supplementation reduced risk by 64% among those with the *rs7968585* genotype and increased risk by 41% among those with one or two G alleles.^(^ ^80^ ^)^ Such work helps identify who may benefit from supplementation for cancer prevention based on vitamin D‐related genotypes. Given the knowledge gap in investigating vitamin D pathway genotypes as modifiers of effects on cancer outcomes, assessment of these in recent trials (possibly as IPD meta‐analyses), and further trials in this research area are both warranted. As recognition of the importance of this, the VITAL trial,^(^ ^62^ ^)^ for example, is in the process of conducting such analyses of gene variants (JE Manson, personal communication, 2020).

## Conclusion

Observational studies showed that, in many cases, low vitamin D was inversely associated with cancer outcomes. For this, the associations for some outcomes, particularly colorectal cancer, seem to fulfill some (but not all) of Bradford‐Hill's criteria for causation,^(^ ^64^ ^)^ including consistency of findings across different meta‐analyses and primary studies, temporality (prospective‐study associations), biological gradient (dose–response associations), and strength of associations (strong in some cases).

To our knowledge, this review is the first report to systematically compile and appraise clinical evidence—by concurrently using AMSTAR‐2, umbrella review, and GRADE assessment tools—of vitamin D supplementation in relation to cancer outcomes from meta‐analyses. We found highly credible RCT evidence that vitamin D supplementation reduces risk of total cancer mortality, but the magnitude of effect was classified as weak. Our finding of a highly credible weak effect on total cancer mortality is in line with that of a 2017 systematic review of meta‐analyses, which reported that vitamin D supplementation reduces risk of all‐cancer mortality.^(^ ^81^ ^)^ We extend that review by including more recent meta‐analyses in our assessment,^13^, ^19^, ^20^, ^21^ ^)^ and by critically evaluating the evidence for this outcome using the above‐mentioned appraisal tools.

The available research, however, is not without limitations. To address these limitations and to provide clearer and further insight into the role of vitamin D in cancer incidence and related mortality, future research should include trials with more vitamin D‐insufficient participants and of longer follow‐up duration, plus adequately powered data on site‐specific cancers (where feasible).

## Disclosures

The authors have no conflicts of interest.

## Author Contributions

**John Sluyter:** Conceptualization; data curation; formal analysis; investigation; methodology; visualization; writing‐original draft; writing‐review and editing. **JoAnne Manson:** Conceptualization; supervision; writing‐review and editing. **Robert Scragg:** Conceptualization; supervision; validation; writing‐review and editing.

### Peer Review

The peer review history for this article is available at https://publons.com/publon/10.1002/jbm4.10420.
